# Chronic Oral Selegiline Treatment Mitigates Age-Related Hearing Loss in BALB/c Mice

**DOI:** 10.3390/ijms22062853

**Published:** 2021-03-11

**Authors:** Judit Szepesy, Viktória Humli, János Farkas, Ildikó Miklya, Júlia Tímár, Tamás Tábi, Anita Gáborján, Gábor Polony, Ágnes Szirmai, László Tamás, László Köles, Elek Sylvester Vizi, Tibor Zelles

**Affiliations:** 1 Department of Pharmacology and Pharmacotherapy, Semmelweis University, H-1089 Budapest, Hungary; szepesy.judit@med.semmelweis-univ.hu (J.S.); humli.viktoria@med.semmelweis-univ.hu (V.H.); farkas.janos2@med.semmelweis-univ.hu (J.F.); miklya.ildiko@med.semmelweis-univ.hu (I.M.); timar.julia@med.semmelweis-univ.hu (J.T.); koles.laszlo@med.semmelweis-univ.hu (L.K.); esvizi@koki.mta.hu (E.S.V.); 2 Department of Otorhinolaryngology, Head and Neck Surgery, Semmelweis University, H-1083 Budapest, Hungary; gaborjan.anita@gmail.com (A.G.); polony.gabor@med.semmelweis-univ.hu (G.P.); szirmai.agnes@med.semmelweis-univ.hu (Á.S.); tamas.laszlo@med.semmelweis-univ.hu (L.T.); 3 Department of Pharmacodynamics, Semmelweis University, H-1089 Budapest, Hungary; tabi.tamas@pharma.semmelweis-univ.hu; 4 Department of Oral Biology, Semmelweis University, H-1089 Budapest, Hungary; 5 Laboratory of Molecular Pharmacology, Institute of Experimental Medicine, H-1083 Budapest, Hungary

**Keywords:** age-related hearing loss, selegiline, chronic oral treatment, hearing protection, mouse model

## Abstract

Age-related hearing loss (ARHL), a sensorineural hearing loss of multifactorial origin, increases its prevalence in aging societies. Besides hearing aids and cochlear implants, there is no FDA approved efficient pharmacotherapy to either cure or prevent ARHL. We hypothesized that selegiline, an antiparkinsonian drug, could be a promising candidate for the treatment due to its complex neuroprotective, antioxidant, antiapoptotic, and dopaminergic neurotransmission enhancing effects. We monitored by repeated Auditory Brainstem Response (ABR) measurements the effect of chronic per os selegiline administration on the hearing function in BALB/c and DBA/2J mice, which strains exhibit moderate and rapid progressive high frequency hearing loss, respectively. The treatments were started at 1 month of age and lasted until almost a year and 5 months of age, respectively. In BALB/c mice, 4 mg/kg selegiline significantly mitigated the progression of ARHL at higher frequencies. Used in a wide dose range (0.15–45 mg/kg), selegiline had no effect in DBA/2J mice. Our results suggest that selegiline can partially preserve the hearing in certain forms of ARHL by alleviating its development. It might also be otoprotective in other mammals or humans.

## 1. Introduction

In line with the globally increasing life expectancy, prevalence of aging-associated diseases and their health care costs are also increasing. The main age-related disorders are Alzheimer’s-disease, stroke, cancer, and atherosclerosis; however, the risk of age-related hearing loss (ARHL) rises as well.

ARHL, also known as presbycusis, is the most common form of sensorineural hearing losses (SNHLs), the prevalence of which is increasing [1]. According to the World Health Organization (WHO), approximately one in three people over the age of 65 years suffer from a certain degree of hearing loss [2]. Due to a decline in hearing ability and speech understanding in noisy environments [3], ARHL threatens personal autonomy, resulting in major difficulties in daily life and, ultimately, social isolation and depression [4].

Underlying factors of cochlear aging include genetic susceptibility, otological disorders, and environmental factors, for example, increased noise exposure [5,6]. The main pathological processes presumed to play a crucial role in the development of ARHL are ischemia, excitotoxicity [6], increased level of reactive oxygen species (ROS) [7], apoptosis [8], and low-grade inflammation [9,10]. As a result, age-related degeneration of stria vascularis, auditory hair cells (HCs), and spiral ganglion neurons (SGNs) could be primarily observed [6,11,12].

The pathophysiology and the genetic architecture of ARHL are generally investigated in different inbred mouse strains due to the fact that mice possess cochlear anatomy [13], physiology, pathophysiology [13,14], and a pattern of ARHL [5,15] similar to humans. In addition, there are many strains of mice with different vulnerabilities to ARHL due to divergent genetic backgrounds [5,8].

Different lines of BALB/c and DBA/2J mice are widely used as murine models in ARHL research [16]. Both strains exhibit the characteristic patterns of human presbycusis [16,17] such as age-related elevation of hearing thresholds beginning at higher frequencies, degeneration of outer hair cells (OHCs) and SGNs beginning at basal cochlear regions, and furthermore, less severe loss of inner hair cells (IHCs) [16]. However, strain-specific variation can be observed in the development of ARHL. In DBA/2J mice, hearing loss progresses more rapidly due to the presence of multiple ARHL-related genes [16].

Although various hearing aids and cochlear implants have been proven to be effective therapies in certain clinical cases, due to its high prevalence and lack of specific pharmacological treatment, ARHL represents an unmet clinical need. Current pharmacotherapeutic approaches in ARHL research focus on testing potential otoprotective drug agents primarily with antioxidant, antiapoptotic or neuroprotective effects, reviewed by Jing Wang and Jean-Luc Puel [8]. Since current drug development programs have not reached phase 3 clinical trials according to (ClinicalTrials.gov; accessed on 24 January 2021) and EudraCT databases, there is still room for exploring novel therapeutical avenues.

Selegiline [(−)deprenyl], a selective and irreversible inhibitor of monoamine oxidase B (MAO-B) [18], was approved for the treatment of Parkinson disease and major depressive disorder [19] by the Food and Drug Administration (FDA) decades ago. Selegiline increases the level of catecholamines; furthermore, neuroprotective, antioxidant, and antiapoptotic effects of this compound has been evidenced as well [20,21]. These properties make selegiline a promising candidate for the treatment of different forms of SNHLs including ARHL. Although the idea of otoprotection in mammals by selegiline was raised and patented (US5561163, EP 0 831 798 B1), it is based on the generalization of the result of a moderately controlled, not-thorough study on outpatient elderly dogs. The study lacked a control group, their hearing was assessed by inadequate behavioral response to sounds such as command and owners’ acknowledgments, and it lasted 1 to 3 months for different dogs [22]. An accurate examination of the potential otoprotective effect of selegiline in ARHL is still missing.

The aim of the present study was to perform a comprehensive investigation of the efficacy of selegiline in preventing or mitigating the deterioration of hearing by age. Here we show that chronic administration of selegiline until the age of week 49 (~1 year) in the dose of 4 mg/kg reduced the progression of ARHL in BALB/c, but not in DBA/2J mice.

## 2. Results

### 2.1. Effect of Chronic Oral Administration of Selegiline on Hearing Function in BALB/c and DBA/2J Mice

**Figure 1:**
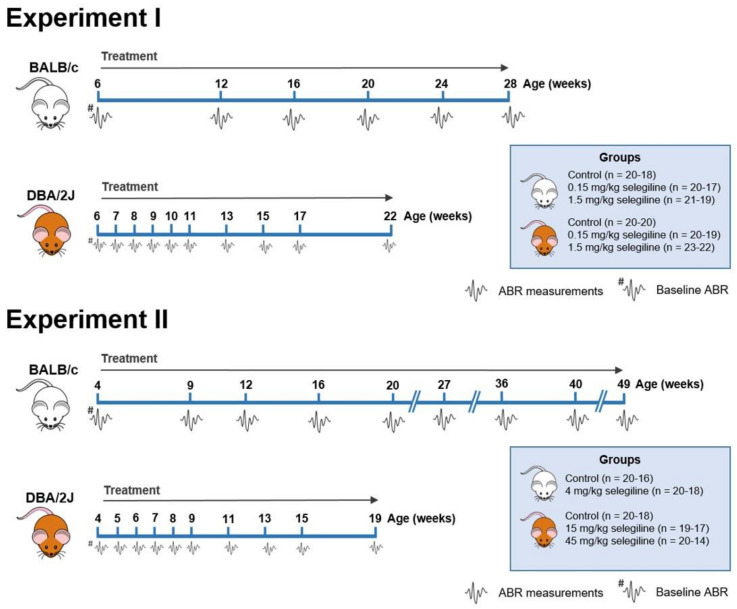
Flow chart showing the treatment protocol and time points of Auditory Brainstem Response (ABR) measurements in BALB/c and DBA/2J mice. Selegiline was dissolved in tap water and freely available for the mice throughout the entire experiment. The daily dose of selegiline was set to a given value (0.15, 1.5, 4, 15, and 45 mg/kg). ABR measurements are indicated by the tiny waveforms. The first ABR measurement (baseline hearing threshold) was performed one day before the onset of selegiline administration. The whole study was carried out in two subsets. The insets show the treatment groups. The number of mice at the start and at the end of the experiments is indicated in parentheses. Experiment I. 0.15 and 1.5 mg/kg selegiline were administered to both BALB/c and DBA/2J mice. The control group received tap water, the solvent of selegiline. In the case of DBA/2J mice, ABR measurements were performed more frequently at the beginning of the experimental period. Experiment II. 4 mg/kg of selegiline was administered to BALB/c mice, and 15 and 45 mg/kg doses to DBA/2J. The dose reduction in BALB/c mice and omission of the 4th treatment group were necessary because this strain lessened its water intake at higher concentrations of selegiline in tap water.

Hearing thresholds of mice were measured at different frequencies and time points to investigate the effect of different doses of selegiline on ARHL in BALB/c and DBA/2J mice. The experimental protocol is presented in Figure 1. See Section 4.3 for the details of the experimental design of auditory measurements and drug administration.

In Experiment II, selegiline, in concentration calculated for ingesting the maximum target dose of 45 mg/kg, caused a substantial reduction in drinking volume to 0.58 mL/mouse/day in BALB/c mice. Testing different concentrations of selegiline in the tap water in parallel with the measurement of water consumption, we chose the 0.05 mg/mL concentration providing an average daily fluid intake of approximately 2 mL/mouse with a 4 mg/kg dose of selegiline.

#### 2.1.1. Experiment I (0.15 and 1.5 mg/kg Selegiline)

**Figure 2:**
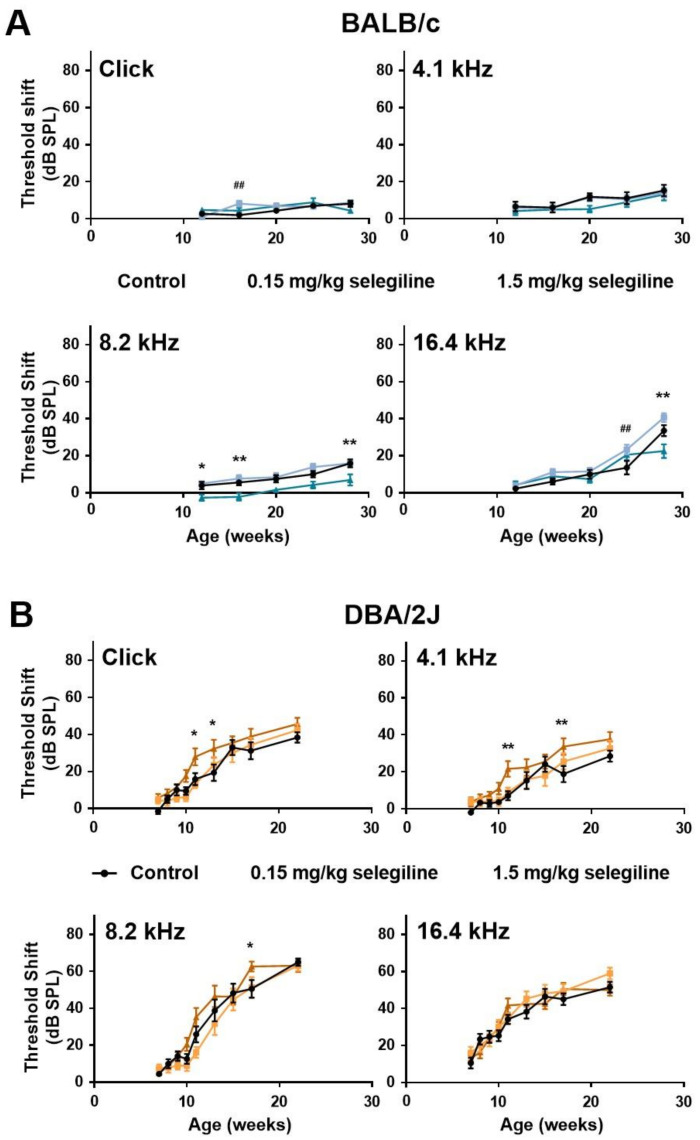
Effect of chronic oral selegiline administration on age-related hearing loss in BALB/c and DBA/2J mice. The drug was added to drinking water. The hearing function was followed by repeated ABR measurements in both the click and tone burst (Figure 1). Treatment of BALB/c (**A**) and DBA/2J (**B**) mice with 0.15 and 1.5 mg/kg selegiline. Data represents mean ± SEM. Two-way ANOVA followed by Bonferroni post-hoc test. 0.15 mg/kg (## *p* < 0.01) and 1.5 mg/kg (* *p* < 0.05, ** *p* < 0.01) selegiline vs. control (see Section 4).

In control BALB/c mice, hearing thresholds progressed gradually with age at all measured frequencies. Auditory threshold shift at 28 weeks of age was 15.29 ± 3.11 dB, 15.88 ± 2.11 dB, and 33.53 ± 2.96 dB at 4.1, 8.2 kHz and 16.4 kHz, respectively, whereas no change was detected using click stimulus (Figure 2A). 0.15 mg/kg selegiline did not influence the thresholds, except enhancements at 16 weeks of age with the click stimulus and at 24 weeks of age at 16 kHz, which seemed rather incidental. A small, but tendentious decrease of the threshold shifts was detected at the dose of 1.5 mg/kg at 8.2 kHz with statistically significant values at ages of 12, 16, and 28 weeks. A similar decrease was measured at the last measuring age, week 28, at 16 kHz (Figure 2A).

In DBA/2J mice, early-onset hearing loss could be observed both with click stimulus and pure tones of different frequencies (Figure 2B). Average threshold shift values were similar in control and 0.15 mg/kg selegiline-treated animals at all time points and measured frequencies, and the same observation applies to click stimulus. Surprisingly, 1.5 mg/kg selegiline enhanced the threshold shifts at 4.1 and 8.2 kHz as well as at click stimulus significantly at some ages (Figure 2B).

These data show that 1.5 mg/kg selegiline has a small but significant protective effect at 8.2 kHz on ARHL in BALB/c mice. In contrast, this dose has rather potentiated the age-dependent threshold shift elevation in DBA/2J mice.

#### 2.1.2. Experiment II (4 and 15, 45 mg/kg Selegiline)

**Figure 3:**
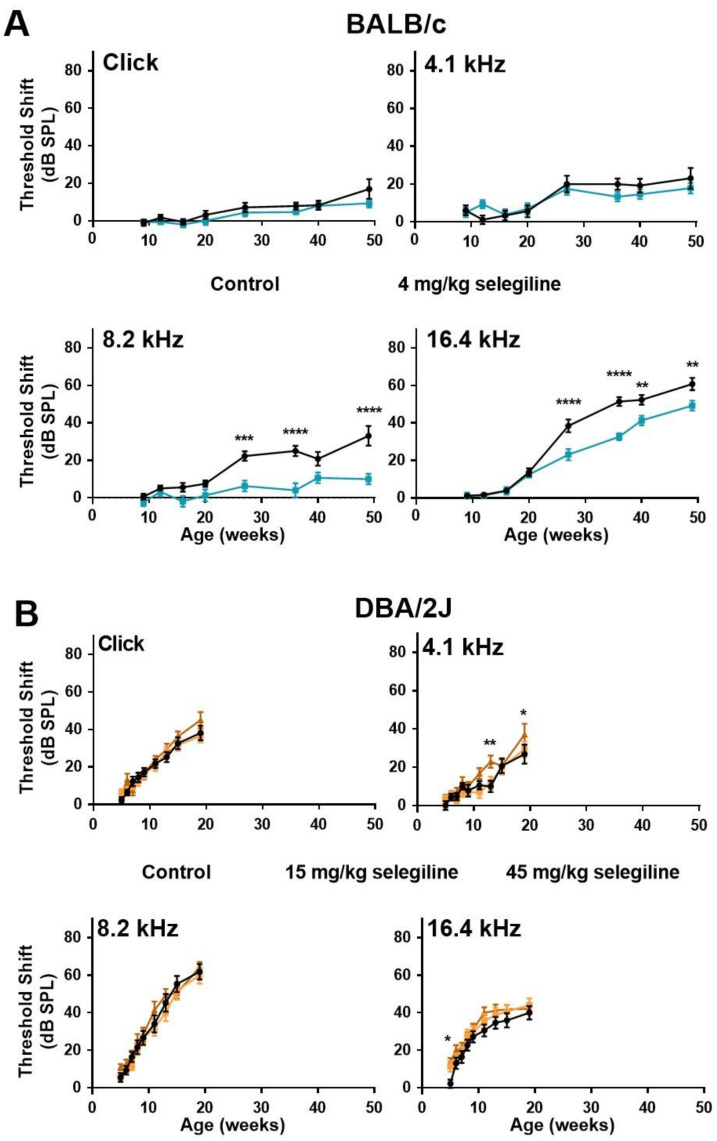
Higher doses of chronic oral selegiline administration alleviated the age-related hearing loss in BALB/c, but not in DBA/2J mice. The drug was added to drinking water. The hearing function was followed by repeated ABR measurements during the age of 4–49 weeks in BALB/c and 4-19 weeks in DBA/2J mice (see protocol in Figure 1). (**A**) Administration of 4 mg/kg selegiline to BALB/c mice. (**B**) Treatment of DBA/2J mice with 15 and 45 mg/kg selegiline. Data represents mean ± SEM. Two-way ANOVA followed by Bonferroni post-hoc test. 4 or 45 mg/kg selegiline vs. control in BALB/c and DBA/2J mice, respectively (* *p* < 0.05, ** *p* < 0.01, *** *p* < 0.001, **** *p* < 0.0001; see Section 4).

In control BALB/c mice, ABR thresholds gradually increased with age at both click stimulus and the three test frequencies (Figure 3A). The highest threshold shift was detected at 16.4 kHz. In the 4 mg/kg selegiline-treated group, threshold shifts at click and at 4.1 kHz were nearly identical to control values during the whole experiment (almost 12 months). At 8.2 and 16.4 kHz, a significant decrease in the threshold shifts was seen after selegiline administration from the 27th weeks of age, compared to the control (Figure 3A).

ABR testing of DBA/2J mice was more frequent at the beginning and covered a shorter time window because of the highly accelerated ARHL in this strain. In these mice, the degree of hearing loss was nearly identical in control and selegiline-treated animals (Figure 3B). Small, but significant elevations appeared at three time points for 45 mg/kg selegiline (at 13 and 19 weeks of age at 4.1 kHz, and at 5 weeks of age at 16 kHz).

These data show that chronic oral administration of 4 mg/kg selegiline significantly alleviated the progressive elevation of hearing thresholds from the age of 27 weeks in BALB/c mice at higher frequencies, while even significantly higher doses failed to influence the progression relevantly in DBA/2J mice.

### 2.2. Effect of Chronic Oral Selegiline Administration on Water Intake, Body Weight and Survival Rate of BALB/c and DBA/2J Mice

#### 2.2.1. Changes in Water Intake

**Figure 4:**
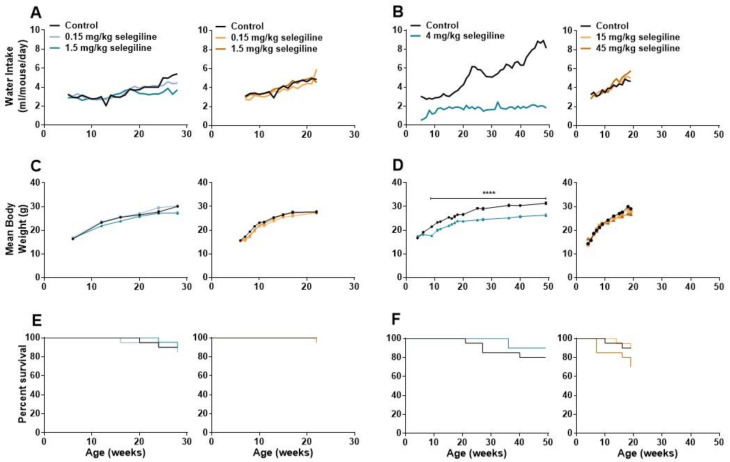
Changes in water consumption, body weight, and analysis of survival during the long term oral treatment by different concentrations of selegiline in BALB/c and DBA/2J mice. Selegiline was administered in tap water. The water intake/day was measured for a whole cage of 10 mice, and the ml/mouse/day values were calculated from that. (**A**) Effect of 0.15 and 1.5 mg/kg (Experiment I) and (**B**) 4 mg/kg and 15 and 45 mg/kg selegiline (Experiment II) on the water intake in BALB/c and DBA/2J mice. (**C**) Effect of the lower (0.15 and 1.5 mg/kg; Experiment I) and (**D**) higher (4 mg/kg and 15 and 45 mg/kg; Experiment II) doses of selegiline on weight gain in BALB/c and DBA/2J mice (**** *p* < 0.0001). (**E**,**F**) The Kaplan-Meier plots show the effect of different doses of per os selegiline on survival rate in BALB/c and DBA/2J mice (compared to control by Mantel-Cox and Gehan-Breslow-Wilcoxon tests; see details in Methods).

Lower doses of selegiline caused a slight decrease in water intake of BALB/c mice in about the last third of the 22-week treatment period. At 28 weeks of age, average fluid consumption of the 0.15 and 1.5 mg/kg selegiline-treated mice was 4.44 and 3.72 mL/day, compared to 5.40 mL/day water intake of control animals. In DBA/2J mice the fluid consumption was similar in all experimental groups (Figure 4A, Experiment I). Statistical analysis of data was not feasible because of the group-housing of mice (10 mice per cage, see Section 4.3).

In Experiment II, water intake of control BALB/c mice was gradually increased during the 4–49 weeks of age experimental period from a daily intake of 2.74–3.31 mL to 8.12–8.85 mL. In contrast, the average daily intake of the 4 mg/kg selegiline-treated group was 1.61–2.14 mL during the entire treatment period. Despite this difference in fluid intake, both experimental groups were in a good general condition. In DBA/2J mice, average fluid consumption was similar in all experimental groups until about the 9th week of treatment, when mice treated with 45 mg/kg selegiline tended to consume more fluid than control animals (Figure 4B, Experiment II).

#### 2.2.2. Changes in Body Weight

Body weights of mice were measured regularly during both Experiment I and II. The control group of BALB/c mice in Experiment I showed a weight gain from 16.40 ± 0.18 g (6 weeks of age) to 30.11 ± 0.34 g (28 weeks of age). Treatment of 1.5 mg/kg selegiline caused a slight, but significant reduction in weight gain (*p* < 0.01 at ages of 12 and 16 weeks and *p* < 0.0001 at age 28 week). 0.15 mg/kg selegiline had no effect. The average weight of control DBA/2J mice increased from 15.7 ± 0.26 g to 27.70 ± 0.51 g (from age of 6 to 22 weeks). Drinking of 0.15 mg/kg selegiline resulted in a slight, but significant reduction in weight gain (*p* < 0.05–*p* < 0.0001 between ages of 7 to 17 weeks). Despite the statistical significance, this weight gain fits well into the range of normal weight gain in this substrain [23] (Figure 4C, Experiment I).

Weight of control BALB/c mice in Experiment II increased from 16.70 ± 0.30 g (4 weeks of age) to 31.37 ± 0.51 g (49 weeks of age). Four mg/kg selegiline treatment significantly reduced the gain of body weight compared to the control during the entire experiment (17.53 ± 0.32 g at 4 and 26.35 ± 0.49 g at 49 weeks of age). The difference was in the 10–18% range, which is in accordance with the ethical guidelines on animal experimentation [24,25,26]. This gain of weight in the selegiline-treated group fits into the range of normal weight gain represented on the growth chart of 3 to 15 week-old BALB/cAnNCrl mice of Charles River Laboratories [23] from where these animals were purchased. Moreover, selegiline-treated mice did not exhibit any signs of pain or distress. The appearance and the natural behavior of the animals were normal during the entire period of the experiment. This reduction could be explained by the avoidance of drinking due to taste preferences in BALB/c mice [27]. In Experiment II, the body weight of control mice increased from 14.40 ± 0.54 g (4 weeks of age) to 29.11 ± 0.43 g (19 weeks of age) in the DBA/2J strain. The rate of weight gain of the 15 mg/kg selegiline treated group, 14.00 ± 0.63 g to 28.12 ± 0.55 g, did not differ from that of the control. Daily oral administration of 45 mg/kg selegiline resulted in a significant decrease of weight gain in time points between the 13th to the 19th week of age (*p* < 0.05–*p* < 0.001). Overall, body weight of this treatment group increased from 16.50 ± 0.52 g to 26.86 ± 0.65 g (Figure 4D, Experiment II).

#### 2.2.3. Survival Rate

As shown in Figure 4E, the survival rates in BALB/c mice were similar in all experimental groups. There was no significant difference between control (90%) and the 0.15 or the 1.5 mg/kg selegiline-treated mice (85% and 90.5%, respectively) at 28 weeks of age (Kaplan-Meier test with log rank (Mantel-Cox) and the Gehan-Breslow-Wilcoxon tests). DBA/2J mice treated with 0.15 mg/kg and 1.5 mg/kg selegiline showed a survival rate of 95% and 95.7% at 22 weeks of age, respectively, while all animals survived in the control group. These results showed no beneficial effect of chronic oral treatment of 0.15 or 1.5 mg/kg selegiline on survival in either mouse strains.

In Experiment II, the survival rate of 4 mg/kg selegiline-treated BALB/c mice was 90% following 45 weeks of treatment and showed no significant difference compared to control mice with a survival rate of 80% (Figure 4F). In DBA/2J mice, the portion of survival was 90%, and 15 mg/kg selegiline treatment did not affect that (89.5%). Mice treated with 45 mg/kg selegiline exhibited only 70% survival at the end of the experiment with no significant difference compared to the other two groups. Although, selegiline administration did not prolong the survival of BALB/c and DBA/2J mouse strains significantly, a slight increase in the survival rate in BALB/c mice and a moderate decrease in the survival in DBA/2J mice with the highest used doses might be observed.

### 2.3. Effect of 4 mg/kg Selegiline on Locomotor Activity

**Figure 5:**
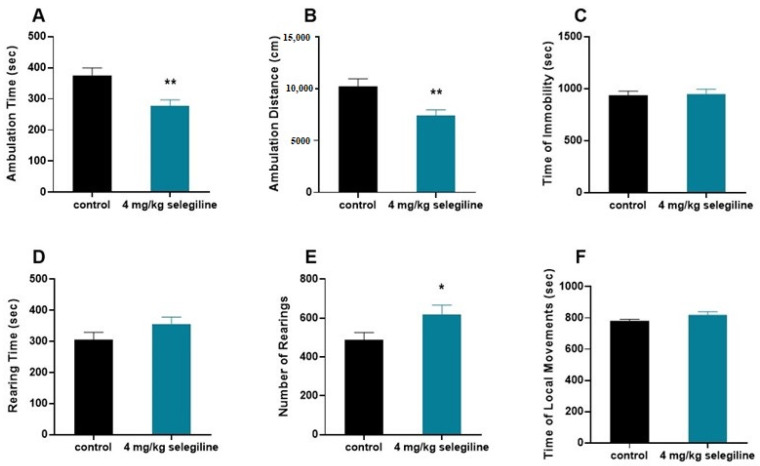
Effect of selegiline (4 mg/kg) on various patterns of locomotor activity of BALB/c mice. The horizontal activity (ambulation; (**A**,**B**)), the vertical activity (**D**,**E**), and the immobility time and local movement time (**C**,**F**) were tested. The observation period lasted 40 min. The control group received tap water (*n* = 9). The treatment group received 4 mg/kg selegiline dissolved in their drinking water (*n* = 9). All data are presented as mean ± S.E.M. Unpaired *t*-test, * *p* < 0.05, ** *p* < 0.01.

We tested the otoprotective dose of selegiline (4 mg/kg) on the behavior of BALB/c mice (Figure 5). The horizontal activity (ambulation) decreased (A–B), while the vertical activity was enhanced (D–E). In general, there was no change in the total activity indicated by the lack of difference in the immobility time and local movement time (C–F).

## 3. Discussion

Specific pharmacotherapy for ARHL is still missing. A number of animal studies have found that targeting the factors involved in the pathomechanism [6,28,29] can be a promising therapeutic direction. Antioxidant therapy, such as administration of N-acetylcysteine [30,31], application of apoptosis inhibitors like X-Linked Inhibitor of Apoptotic Protein [32], or neuroprotective compounds [8], have a protective effect on ARHL, but none of these drugs were involved in phase 3 clinical trials according to (ClinicalTrials.gov; accessed on 24 January 2021) and EudraCT databases. The FDA-approved antiparkinsonian drug selegiline is known as an anti-aging drug [33], has complex neuroprotective, antioxidant, and antiapoptotic effects [34,35,36]. Therefore, we considered it relevant to examine whether selegiline shows a positive impact on presbycusis and lessen the progression of the disorder.

The potential otoprotective effect of different doses of selegiline on ARHL was tested in two mouse strains. BALB/c mice show a massive age-related decline in auditory functions by the age of 10 months [16,17], but age-related changes in the auditory function of this strain gradually increase from 4 weeks of age, primarily at higher frequencies, and lead to clearly noticeable elevation of hearing threshold values from the age of 24–28 weeks. In DBA/2J mice, age-related hearing impairment already begins after weaning, and this strain exhibits severe loss of auditory functions by 12 weeks of age [16,17,37].

Since individual differences in the time course of ARHL also occur in humans, investigation of potential otoprotective drug candidates against ARHL in mouse strains with different progression of hearing loss increases the translational value of findings. In addition, involvement of two different mouse strains improves the generalizability of study results [38].

Based on the progression rate of hearing loss, we considered that chronic administration of selegiline from a young age might be more beneficial. Administration of selegiline at the dose of 4 mg/kg alleviated the progression of ARHL in BALB/c mice. This protection was pronounced at higher frequencies from the age of 27 weeks, including the most sensitive frequency range of mice [39], and preserved throughout the experiment. In contrast, the protective effect of selegiline cannot be observed in DBA/2J mice. BALB/c strain is homozygous for the Ahl1 allele, while the larger susceptibility of DBA/2J strain for ARHL is due to the presence of Ahl1, Ahl8, and Ahl9 genes [40,41,42]. Differences in the efficacy of selegiline would presumably be due to the presence of more ARHL predisposing genes in DBA/2J strain, which might cause the higher progression rate, severity, and probably a more complex pathology leading to ARHL.

The question arises which beneficial properties of selegiline might be behind the otoprotective effect. It is known that degeneration of outer hair cells (OHCs) and spiral ganglion neurons (SGNs) is one of the main characteristic patterns of ARHL [16]. In DBA/2J mice, age-dependent loss of OHCs and SGNs are extremely severe and occur already in young mice [16]. Early loss of auditory function in this strain is most likely associated with early degeneration of OHCs [16]. On the contrary, in the BALB/c strain, loss of SGNs begins after 4 months and progresses gradually [16,43,44]. After several weeks, this neural loss may manifest in the elevation of hearing thresholds. Willott et al. found that loss of OHCs starts between 50 days and 4 months of age at the cochlear base. The middle regions are affected less, and only by 10 months [16]. In our experiments, a significant decrease in the progression of the elevation of hearing thresholds in the 4 mg/kg selegiline-treated group was seen at 8.2 and 16.4 kHz from 27 weeks of age. The time of appearance of this protective effect correlates with the time course of SGN loss in BALB/c mice. Therefore, selegiline-induced neuroprotection might be one of the main contributors to its otoprotective effect observed in ARHL.

A further mechanism involved in the otoprotective effect of selegiline might be its dopamine (DA) release enhancing action. The excessive release of glutamate (Glu) from inner hair cells (IHCs) in different forms of SNHLs, including the ARHL, initiates to the excitotoxic damage of the primary auditory neurons and their synapse with the IHCs [45,46,47]. This excitotoxic overactivation is inhibited by DA released from the lateral olivocochlear (LOC) efferents forming axodendritic synapses on the auditory neurons, thereby protecting the IHC-afferent nerve synapse [47,48,49,50,51]. Changes in the cochlear dopaminergic system in aging animals have been previously described. Vicente-Torres et al. reported that the concentration of DA and its metabolites were enhanced in the cochlea in older rats, and this increase could constitute a compensatory mechanism against the age-related loss of afferent type I neurons [52]. Theoretically, any drug that boosts the function of the LOC-DA system could provide preventive or curative effects on ARHL [51,53,54]. Selegiline, through the inhibition of MAO enzyme, affects dopaminergic neuronal transmission and enhances the release of DA in the nervous system [34,55]. Polony et al. showed that rasagiline, a congener of selegiline [35], by blocking the metabolism and uptake of DA, enhanced the release of DA from LOC terminals in mouse cochlear preparation and ameliorated the hearing impairment induced by an aminoglycoside antibiotic [51]. Furthermore, this otoprotective effect might persist during long-term selegiline treatment because of the lack of alteration in the sensitivity of DA receptors [56].

The ineffectiveness of selegiline in DBA/2J strain does not diminish the significance of its otoprotective effect in BALB/c mice. As different subtypes of ARHL are present in different mouse strains of ARHL models, individual genetic predispositions related to age-related auditory degeneration can be observed in humans [57,58,59,60]. This results in subpopulations of treatment-resistant and treatment-responsive patients [8]. Selegiline might show an otoprotective effect in some, but not all of these clusters, depending on the individual genetic background (personalized medicine).

Besides otoprotection, administration of selegiline showed the unexpected effect of reduced water intake and a decreased weight gain of mice with a pronounced presence in the BALB/c substrain.

Selegiline was dissolved in drinking water and administered chronically. This way of drug application eliminates the trauma and also the risk of infections associated with daily parenteral injections or oral gavage. Furthermore, it is the preferred drug delivery route in human patients [61,62]. In several previous studies, selegiline was administered via this route to rodents, and it had no effect on fluid consumption [63,64,65]. In our experiments, contrary to the literature, the administration of selegiline in drinking water led to a reduction in drinking in BALB/c mice in a concentration-dependent manner. The planned doses of 15 or 45 mg/kg could not be reached in the BALB/c mice. On the contrary, decreased fluid consumption could not be observed in the DBA/2J strain. It has been described that the BALB/c6NCrlBL substrain exhibits lower preferences to higher molar concentrations of NaCl, citric acid, and quinine HCl as well [27]. Moreover, BALB/c mice show significant sensitivity to bitter taste [66]. According to a report by the National Toxicology Program, decreased water intake of BALB/c mice relates to the taste of the drinking solution [67]. Based on these findings, we hypothesize that the reduced fluid consumption was related to the special strain specific taste preference of BALB/c mice, i.e., this strain does not like the taste of selegiline-HCl.

There was also a decrease in weight gain in selegiline-treated BALB/c groups. It did not mean a real decrease in body weight, but a restraint on the weight gain that showed a correlation with the concentration of selegiline in the drinking water. Decreased fluid consumption of 4 mg/kg selegiline-treated BALB/c groups occurred even before initiation of the reduction in body weight gain. This may support the hypothesis that decreased weight gain might be the result of the decreased food consumption caused by the compensatory reduction of water intake. Reduced food intake is a protective response of the body to defend the fluid balance [68].

It has been reported that caloric restriction without malnutrition could reduce the severity of ARHL [69,70]. However, results on this topic are contradictory. Sweet et al. reported that caloric restriction could mitigate the progression of ARHL in CBA/J mice if the restriction occurs at the initial phase of degeneration of the auditory system [71]. Effects of dietary restriction on ARHL in different inbred mouse strains were also investigated by Kenneth R. Henry [72]. In AKR mice, which strain shows early-onset hearing impairment, dietary restriction affected neither the life span nor the progression of ARHL. By contrast, AU/Ss mice on a restricted diet lived longer and had less severe ARHL compared to their littermate controls. Henry has emphasized that the relation between cochlear function and dietary restriction is genotype-dependent. Willot at al. found that strain specific ameliorative effects of caloric restriction on age-related cochlear degeneration, if it could be observed at all, are limited [73]. Although we cannot rule out the possibility of caloric restriction based otoprotection in BALB/c mice, contradictory results in the literature and the differences between the time course of the appearance of hearing protection and decreased body weight in our study argue against its potential otoprotective effect. Moreover, a number of studies found that decreased body weight of selegiline treated animals do not contribute to the life prolonging effect of selegiline [63,74,75,76].

Our results of survival analysis were less unexpected. Significant differences in longevity between control and selegiline-treated groups could be observed neither in DBA/2J nor in BALB/c mice. Although life prolonging effects of chronic selegiline treatment have been reported in rats, hamsters, and dogs, a number of studies failed to obtain positive longevity effects in mice [63,77].

The 4 mg/kg protective dose of selegiline in the BALB/c mice, by using the FDA guidance (https://www.fda.gov/media/72309/download; accessed on 24 August 2018) for mouse to human dose conversion, gives an approximate of 20 mg/day human equivalent dose. The use of a higher than human antiparkinsonian dose (5–10 mg/day) of selegiline raises the possibility of enhanced activity, a possible side effect of the drug in higher dose. Our behavioral study on 4 month-old BALB/c mice showing otoprotection for 4 mg/kg selegiline did not substantiate this notion. Though selegiline treatment affected the features of locomotor activity, namely enhanced the initial exploratory behavior (rearing) and in line with this reduced the ambulation, the lack of change in immobility and local movements, however, strongly speaks against a possible activity enhancing action of it. This supports its repositioning in higher dose to delay ARHL progression.

In the present study, we demonstrated that chronic oral administration of selegiline mitigated the development of age-related hearing loss in BALB/c, but not in the DBA/2J mice. Preserved hearing function of BALB/c mice could be explained by the neuroprotective, antiapoptotic, antioxidant, and DA neurotransmission enhancing (LOC) effects of selegiline. However, we cannot exclude the possible otoprotective effect of caloric restriction observed in our experiments. Strain differences indicate that the protective effect of selegiline depends on the host’s genetic background. Direct translation of our results to clinical application would suggest that chronic selegiline treatment seems to be a reasonable therapy in certain types of human ARHL, taking into account individual genetic predisposition (personalized medicine).

## 4. Materials and Methods

### 4.1. Ethics Statement

Animal care and experimental procedures were approved by the National Scientific Ethical Committee on Animal Experimentation and the Semmelweis University’s Institutional Animal Care and Use Committee (H-1089 Budapest, Hungary) and permitted by the Government Office of Pest County Division of Food Chain Safety and Animal Health Directorate (project identification code: PE/EA/1912-7/2017). Mice were handled with the principles of NIH guidelines (National Research Council (2011), Guide for the Care and Use of Laboratory Animals: Eighth Edition).

### 4.2. Experimental Animals and Housing Conditions

Experiments were performed on male BALB/cAnNCrl (#028) and male DBA/2J (#625) mice, hereafter referred to as BALB/c and DBA/2J. Animals were purchased from Charles River’s facilities located in Germany and France, respectively (Charles River Laboratories, Wilmington, Massachusetts, 4 weeks of age at arrival). Animals were housed and maintained under a 12:12 h light–dark cycle and controlled environmental conditions (20–24 °C and 35–75% relative humidity) with ad libitum access to food and water throughout the entire duration of the experiment.

### 4.3. Experimental Design of Hearing Function Measurements and Selegiline Administration

In order to test the effect of a broader selegiline dose range and because of the high number of animals per group and the limits of ABR recordings per day, these measurements were divided into two separate experiments. Chronic administration of selegiline was achieved by adding Selegiline HCl (Chinoin Private Co. Ltd., Budapest, Hungary) to drinking water.

Experiment I. Six-week-old male BALB/c and DBA/2J mice were divided into 3 treatment groups each per strain: BALB/c: Control (*n* = 20–18), selegiline-treated 0.15 mg/kg (*n* = 20–17), and selegiline-treated 1.5 mg/kg (*n* = 21–19). DBA/2J: Control (*n* = 20–20), selegiline-treated 0.15 mg/kg (*n* = 20–19), and selegiline-treated 1.5 mg/kg (*n* = 23–22). The number of mice at the start and at the end of the experiment is indicated in parentheses. BALB/c mice were treated until the age of week 28, and their hearing function was monitored (ABR) regularly. DBA/2J mice were treated and monitored for a shorter time (weeks of age 6–22), because the progression of ARHL is more rapid in this strain, and their mean hearing thresholds at frequencies above 8 kHz are 80–90 dB by that age [16,78].

Selegiline administration was started right after the first measurement of auditory functions and continued until the last measurement of hearing thresholds. Selegiline was dissolved in drinking water (tap water). Body weight of each mouse and water intake of each cage were monitored every 3 days by weighing the mice and water bottles. Ten mice were housed per cage; therefore, individual water intake and oral ingestion of selegiline could not be determined, but per mouse ingestion of selegiline was calculated. The concentration of selegiline in the bottles was adjusted to set and keep the required dose in the actual treatment group. Based on these estimates, the following doses were administered: BALB/c mice (mean ± SD): 0.14 ± 0.05 mg/kg and 1.32 ± 0.41 mg/kg; DBA/2J mice (mean ± SD): 0.19 ± 0.08 mg/kg and 1.91 ± 0.75 mg/kg, referred to henceforth as the 0.15 mg/kg and 1.5 mg/kg doses, respectively. This inevitable variability in dose levels is inherent to the oral administration method, which avoids daily parenteral injections with stress and risk of infections, on the other side. Control animals received regular tap water.

Experiment II. In a second set of experiments, the effect of higher doses of selegiline was investigated. In BALB/c mice the tested period was also extended significantly (weeks of age 4–49).

Four-week-old male BALB/c and DBA/2J mice were divided into the following treatment groups. BALB/c: Control (*n* = 20–16), selegiline-treated 4 mg/kg (*n* = 20–18). DBA/2J: Control (*n* = 20–18), selegiline-treated 15 mg/kg *(n* = 19–17), and selegiline-treated 45 mg/kg (*n* = 20–14). The number of mice at the start and at the end of the experiment is indicated in parentheses. Hearing function was monitored (ABR) regularly. DBA/2J mice were treated and monitored for a shorter time (weeks of age 4–19), because of their more rapid progression of ARHL (Figure 3B).

The housing of mice, the way of selegiline administration, measurement of water consumption and body weight and the calculation and adjustment of selegiline concentration to achieve the required ingestion of the drug were identical to Experiment I. BALB/c mice, but not the DBA/2J strain, reduced their intake from water containing high concentrations of selegiline, and the highest ingested dose we could reach was 4 mg/kg. Therefore, we did not run a 4th treatment group in BALB/c mice. Based on the estimates, the following doses were administered: BALB/c mice (mean ± SD): 3.84 ± 0.55 mg/kg; DBA/2J mice (mean ± SD): 14.93 ± 2.81 mg/kg and 46.82 ± 11.35 mg/kg, referred to henceforth as the 4 mg/kg and 15 and 45 mg/kg doses, respectively. Control animals received regular tap water.

### 4.4. Auditory Brainstem Response (ABR) Recordings

ABR tests were performed to follow up changes in auditory function by measuring hearing thresholds as previously described [9,51]. In brief, mice were anesthetized with a mixture of ketamine-xylazine injection (100 mg/kg and 10 mg/kg, intraperitoneally, respectively). The core temperature of mice was maintained between 36 and 38 °C using a temperature-controlled heating pad (Supertech Instruments, H-7624 Pécs, Hungary). For recording evoked potentials, needle electrodes were placed subcutaneously at the vertex (active electrode), behind the right pinna (reference electrode), and at the rear leg (ground). Hearing tests were performed in an electrically shielded sound-proof chamber using an auditory research system developed by Tucker Davis Technologies (TDT system 3 with RX6 signal processor and RA16 Medusa Base Station; Tucker–Davis Technologies (TDT), Alachua, FL, USA). Auditory stimuli consisting of click (0.4 ms duration, with bandwidths of 0–50 kHz) and 4-, 8-, and 16 kHz tone bursts (3 ms duration, 0.2 ms rise/decay) were digitally generated in the SigGenRP software package (TDT, Alachua, FL, USA) and delivered into the right ear through an EC-1 electrostatic speaker in a closed acoustic system, controlled by the BioSigRP software (TDT, Alachua, FL, USA). All biological signals were amplified through RA4PA Medusa PreAmplifier (TDT, Alachua, FL, USA) connected to RA4LI Low Impedance Headstage (TDT, Alachua, FL, USA). Sound pressure levels (SPL) of the click stimulus were increased in 10-dB steps from 0 to 80 dB. In tone burst stimulation mode, the intensity was attenuated in 10 dB steps from 90 to 10 dB at each frequency. Attenuation was controlled by a PA5 Programmable Attenuator (TDT, Alachua, FL, USA). For calibrating the sound delivery system, a half-inch free field preamplifier integrated microphone was used (ACO Pacific Inc., Belmont, CA 94002, USA; Model 7017) with the application of the SigCalRP (TDT, Alachua, FL, USA) calibration software. Responses were amplified, filtered, and averaged 800 times in real-time. The hearing threshold was defined as the minimal intensity level at which an ABR waveform with an identifiable peak could be detected visually. Shifts in auditory thresholds were calculated for click and tone bursts by subtracting the auditory thresholds registered at the start of the experiment (baseline auditory threshold levels) from the hearing thresholds registered at different ages.

The tested frequency range of 4–16 kHz in mice corresponds approximately to the 1–4 kHz range in human beings [39]. This range is essential for normal speech perception and regularly tested during basic audiologic assessments [79,80,81,82,83], and also used in guidelines recommending the sound pressure level of hearing impairment that is required for prescribing hearing aids [84]. This matching in the practically relevant mouse-human frequency range provides a reliable translational value to our study.

### 4.5. Survival Analysis

Mice in each cage were controlled daily. The effect of different doses of selegiline on the survival of BALB/c and DBA/2J mice was analyzed by the Kaplan-Meier method, and the curves were compared by the log rank (Mantel-Cox) and Gehan-Breslow-Wilcoxon tests. Survival rate was plotted as the percent of survival.

### 4.6. Test of Locomotor Activity

Locomotor activity of some control (*n* = 9) and selegiline-treated (4 mg/kg; *n* = 9) BALB/c mice at their 4 months of age in Experiment II were measured by “CONDUCTA System for behavioral and activity studies” (Experimetria Ltd., H-1062 Budapest, Hungary). The apparatus consists of three black-painted testing boxes (40 × 50 × 50 cm each) set in an isolated room. Three animals could be tested in parallel without any connection between them. One animal was placed in one box. Ambulation (walking, running) time and distance, rearing, local movement, and immobility time were recorded individually for each box. The movements of mice were detected by high-density arrays of infrared diodes. The observation started immediately without any habituation and lasted 40 min. Mice were absolutely naïve to the apparatus, and they were placed into the experimental box only once.

### 4.7. Data Analysis

Change of the auditory thresholds was expressed as a threshold shift. Two-way ANOVA followed by the Bonferroni post-hoc test was performed to determine the statistical significance in the 6.01 version of GraphPad Prism. Calculations were computed separately at every frequency and click stimulation. One-way ANOVA followed by Bonferroni post-hoc test was used to compare body weights between control and selegiline-treated animals. Survival rate differences were analyzed by Kaplan-Meier method with log rank (Mantel-Cox) and Gehan-Breslow-Wilcoxon tests using GraphPad Prism (v.6.01). Analyses of locomotor activity were performed using GraphPad Prism v.8.0.1. Statistical significance of difference between mean values was evaluated by Unpaired Student’s *t*-test. Data are expressed as mean ± standard error of the mean (SEM). For all comparisons, levels of significance are as follows: * *p* < 0.05, ** *p* < 0.01, *** *p* < 0.001, **** *p* < 0.0001.
